# Study of genetic markers of cardiac arrhythmias in Kazakhstan

**DOI:** 10.5195/cajgh.2013.85

**Published:** 2014-01-24

**Authors:** Makhabbat Bekbossynova, Ainur Akilzhanova, Zhannur Abilova, Ayan Abdrahmanov, Omirbek Nuralinov

**Affiliations:** 1 National Research Center for Cardiac Surgery, Astana, Kazakhstan; 2 Department for Organization and Development of Genomic and Personalized Medicine, Center for Life Sciences, Nazarbayev University, Astana, Kazakhstan

**Keywords:** cardiac arrhythmia, genetic screening, genetic markers, Kazakhstan

## Abstract

**Introduction:**

Cardiac arrhythmias are the most common cause of mortality and sudden cardiac death worldwide. In the past decade, genetic factors underlying arrhythmogenic diseases have been revealed and given novel insights in to the understanding and treatment of arrhythmias predisposing one to sudden cardiac death.

**Material and methods:**

We conducted a pilot genetic screening of two patients with catecholaminergic polymorphic ventricular tachycardia (CPVT) and 14 patients with ventricular tachycardia (VT) for genetic variants in the human ryanodine receptor gene 2 (hRYR2). The most relevant 45 hot-spot exons of hRYR2 were amplified by polymerase chain reaction (PCR) and directly sequenced.

**Results:**

One novel mutation in a CPVT patient (c.A13892T; p.D4631V) and a novel mutation in a VT patient (c.G5428C; p.V1810L) were identified. Both variants are located at phylogenetically conserved positions and predicted pathogenesis. Three known synonymous SNPs (rs3765097, rs2253273, and TMP ESp1 237664067) were detected in the study group. No further variants within the target regions were detected in the study group.

**Conclusion:**

The results of study can be applied to risk asssessment for life-threatening arrhythmias and assist in development of appropriate strategies for prevention of sudden cardiac death. The implementation of these strategies would assist in the management of patients with genetically determined arrhythmias in Kazakhstan.

